# Construction of a nomogram prediction model for prolonged ICU length of stay in patients with sepsis-induced coagulopathy

**DOI:** 10.1186/s40001-026-03948-2

**Published:** 2026-01-27

**Authors:** Xinbei Zhang, Hongxia Cai, Xiaomin Zhang, Yao Zhou, Liangzhe Zou, Gaoke Kong, Zhimei Gao, Su Tu

**Affiliations:** 1 Wuxi Medical College, Jiangnan University, Wuxi, China; 2 Emergency Department, Central Hospital Affiliated to Jiangnan University, Wuxi, China

**Keywords:** Sepsis, Sepsis-induced coagulopathy, Length of stay, Nomograms, Risk assessment, Predictors

## Abstract

**Objective:**

To develop and evaluate a nomogram model for predicting prolonged ICU length of stay (LOS) in patients with sepsis-induced coagulopathy (SIC), identify associated risk factors, and facilitate early identification of high-risk patients, with the aim of optimizing clinical management strategies, improving patient outcomes, and enhancing ICU resource utilization.

**Method:**

A total of 3728 patients meeting the diagnostic criteria of International Society for Thrombosis and Hemostasis (ISTH) criteria were included from the Medical Information Mart for Intensive Care (MIMIC-IV) database. Based on the third quartile value of ICU LOS in the cohort, patients were categorized into a prolonged ICU LOS group (≥ 5 days) and a non-prolonged ICU LOS group (< 5 days). General demographic data, clinical characteristics, and laboratory test results within 24 h of ICU admission were collected to identify independent risk factors for prolonged ICU LOS in SIC patients. Predictive variables were selected using LASSO-logistic regression combined with Shapley Additive Explanations (SHAP) for interpretability. A nomogram model was constructed and evaluated using receiver operating characteristic (ROC) curves, calibration curves, and decision curve analysis (DCA).

**Results:**

Among the 3728 enrolled patients, 832 had a prolonged ICU LOS (≥ 5 days), while 2896 had a non-prolonged ICU LOS (< 5 days). LASSO-logistic regression and SHAP analysis identified six predictive variables: SOFA score, heart rate, monocyte percentage, acute kidney injury (AKI), use of vasopressors, and use of mechanical ventilation. The nomogram demonstrated an area under the curve (AUC) of 0.737 (95% CI 0.716–0.758).

**Conclusion:**

Risk factors for prolonged LOS in patients with SIC included: increased SOFA score, elevated heart rate, higher monocyte percentage, occurrence of AKI, use of vasopressors, and use of mechanical ventilation. By integrating these readily available clinical indicators into an intuitive nomogram, we have developed a practical risk assessment tool for clinicians. This tool aids in the identification of SIC patients at risk for prolonged hospitalization. Furthermore, our analysis revealed that prolonged LOS was significantly associated with increased long-term mortality. The application of this predictive model may ultimately contribute to reducing ICU length of stay and improving patient prognosis.

## Introduction

Sepsis is a life-threatening organ dysfunction syndrome triggered by a dysregulated host response to infection [1], frequently accompanied by hemodynamic instability and metabolic disturbances. As a disease characterized by high incidence, mortality, and treatment costs, sepsis accounts for approximately 20% of global deaths [2]. Concurrently, sepsis stands as the most common cause of admission to intensive care units (ICU) and the leading contributor to ICU mortality [3]. One study reports an ICU mortality rate of approximately 40% among sepsis patients [4].

Coagulation abnormalities are nearly universal in septic patients, involving pathological processes such as the release of inflammatory mediators, endothelial injury, and platelet dysfunction, culminating in sepsis-induced coagulopathy (SIC) [5]. SIC is characterized by thrombocytopenia, prolonged clotting times, and excessive fibrin formation [6]. As a common complication of sepsis, SIC occurs in approximately 24% of cases [7]. If not promptly managed, it can progress to disseminated intravascular coagulation (DIC), doubling patient mortality [8]. Current research on the ICU length of stay (LOS) for SIC patients remains relatively limited. Several scholars propose average LOS as a key metric for evaluating hospital operational efficiency and resource utilization [9]. Models integrating admission data and laboratory parameters to predict ICU LOS can facilitate early clinical intervention, cost control, and prevention of disease progression to DIC, thereby improving patient outcomes and guiding clinical decision-making. Studies indicate that higher SIC scores correlate with increased sepsis mortality, suggesting that prolonged ICU LOS leads to higher hospitalization costs and worse prognoses [10].

In recent years, nomograms have emerged as increasingly valuable predictive tools in clinical decision-making, aiding healthcare providers in navigating clinical scenarios. They represent graphical calculators that visualize the underlying statistical model, allowing for the direct calculation of outcome probabilities. Within a nomogram, the length of each scale corresponds to the importance of each parameter in predicting the outcome, a longer scale indicates greater significance of that variable [11, 12]. It has been applied to various related diseases, including intoxication [13] and SIC [14], among others. To further enhance interpretability, the Shapley Additive Explanations (SHAP) algorithm was employed. This algorithm quantifies the contribution of each predictive variable to the model’s output, thereby clarifying the positive or negative influence of individual features on patient prognosis [15]. In addition, we also evaluated the association between prolonged ICU stay and mortality.

Currently, no risk prediction model has been established specifically for patients with Sepsis-Induced Coagulopathy (SIC). Given that SIC is a severe complication of sepsis, its management and prognosis directly impact patient recovery and healthcare resource allocation. Therefore, this study aims to investigate the risk factors associated with prolonged ICU stay in SIC patients and to develop a predictive model using easily obtainable clinical data from the early ICU admission period to assess the likelihood of extended ICU length of stay, supporting clinicians in early risk stratification and personalized treatment.

## Methods

### Data source

This study was a retrospective cohort analysis. Data were extracted from the Medical Information Mart for Intensive Care IV (MIMIC-IV, Version 3.1), a large, publicly available single-center critical care database developed through collaboration between Beth Israel Deaconess Medical Center (BIDMC) and the Massachusetts Institute of Technology (MIT). The study was carried out in accordance with the Declaration of Helsinki. Because the MIMIC repository contains de-identified data, no sensitive information was involved. Thus, this article does not require an ethics application, and the author of this article has been approved to access the MIMIC-IV database and has completed the required training.

### Patient diagnosis and selection

**Fig. 1:**
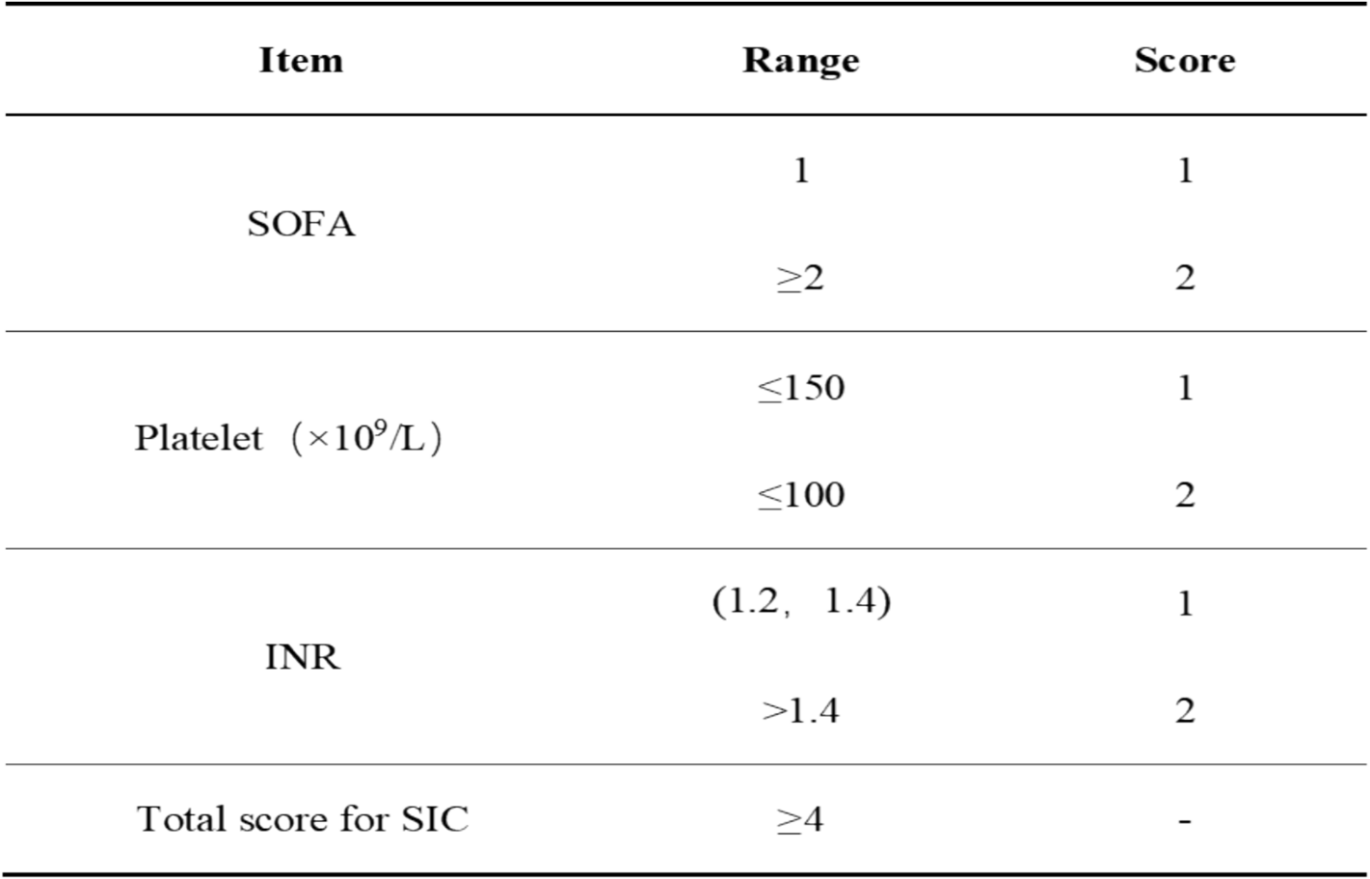
ISTH criteria for SIC diagnosis [16]. SOFA, Sequential Organ Failure Assessment; INR, international normalized ratio; SIC, sepsis-induced coagulopathy

Currently, there is no gold standard for diagnosing SIC. The diagnostic criteria for SIC adopted in this study were proposed by the International Society for Thrombosis and Hemostasis (ISTH) [16], consisting of three indicators: SOFA score, platelets, and the international normalized ratio of INR. A score of ≥ 4 can be diagnosed as SIC (Fig. 1).

### Inclusion and exclusion criteria

**Fig. 2:**
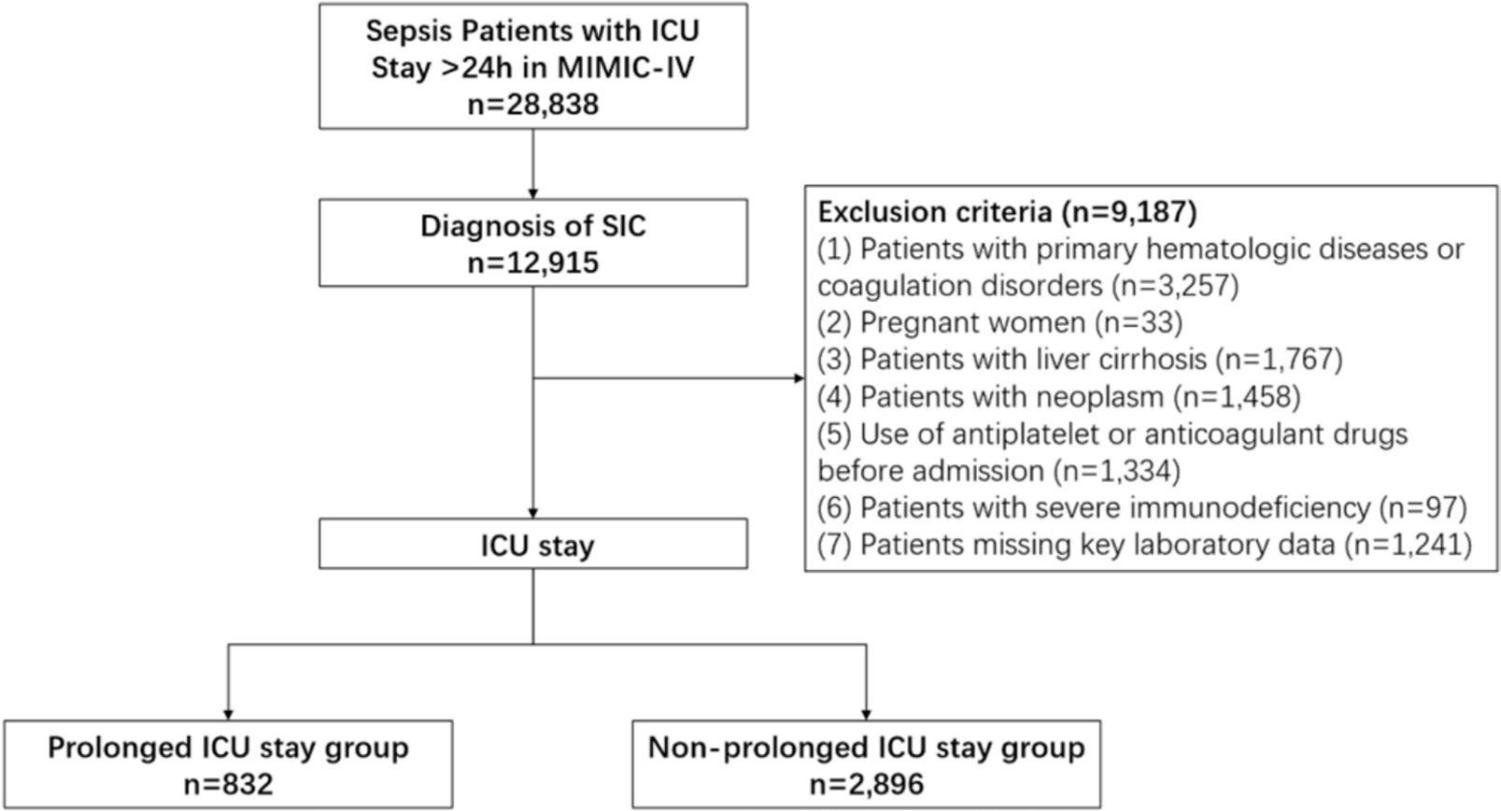
Patient screening flow

The inclusion criteria for this study are: (1) age ≥ 18 years; (2) first ICU admission lasting ≥ 24 h; (3) meeting the Sepsis-3.0 criteria [17] ; (4) SIC score ≥ 4 on the first day post-ICU admission. Exclusion criteria: (1) patients with primary hematologic diseases or coagulation disorders; (2) pregnant women; (3) patients with liver cirrhosis; (4) patients with neoplasm; (5) use of antiplatelet or anticoagulant drugs before admission; (6) patients with severe immunodeficiency; (7) patients missing key laboratory data (Fig. 2).

### Data collection

The following information was extracted from the MIMIC-IV database using the PostgreSQL and Navicat 16.1.12 database management systems: (1) demographic information: gender, age, height, weight, and body mass index (BMI); (2) basic vital signs (first recorded within the initial 24 h): temperature, heart rate, respiratory rate, systolic blood pressure (SBP), diastolic blood pressure (DBP), mean arterial pressure (MAP), and oxygen saturation (SpO₂); (3) disease severity scores (within the initial 24 h): Glasgow Coma Scale (GCS) score and Sequential Organ Failure Assessment (SOFA) score; (4) laboratory parameters (within the first 24 h following ICU admission): red blood cell count (RBC), white blood cell count (WBC), platelet count (PLT), red cell distribution width (RDW), international normalized ratio (INR), neutrophil percentage, lymphocyte percentage, and monocyte percentage; (5) treatment modalities (within the initial 24 h): use of mechanical ventilation and use of vasopressors; (6) underlying diseases/comorbidities: hypertension, type 2 diabetes mellitus (T2DM), acute kidney injury (AKI), and heart failure (HF); (7) length of stay in the intensive care unit (ICU); (8) clinical outcomes: mortality at 7, 14, 28, and 90 days post-admission.

### Missing data handling

Variables with ≥ 30% missing data were excluded. Variables with < 30% missing data were imputed using multiple imputation in SPSS 25.0 to minimize bias.

### Statistical analysis

This retrospective cohort study employed SPSS 25.0 and R 4.4.2 for statistical analysis. Missing data were addressed using multiple imputation methods. Categorical and continuous variables were presented as frequencies (percentages) and mean ± standard deviation (SD), respectively. Between-group comparisons were performed using Student’s *t*-test, Chi-square test, or non-parametric alternatives (such as the Mann–Whitney *U* test or Kruskal–Wallis test). For non-normally distributed variables, the Kruskal–Wallis test was applied, with results reported as median and interquartile range (IQR). Categorical variables were expressed as percentages, with specific test selection based on data distribution and underlying assumptions to ensure analytical reliability. Variable selection and regularization were conducted using Least Absolute Shrinkage and Selection Operator (LASSO)-logistic regression to identify key variables and mitigate multicollinearity in multivariate model fitting. Subsequently, the Shapley Additive Explanations (SHAP) method was employed to quantify the predictive contribution of each retained variable, thereby simplifying the model. A nomogram was then constructed to predict prolonged ICU length of stay in patients with sepsis-induced coagulopathy (SIC). A comprehensive evaluation approach was utilized to assess model performance, encompassing receiver operating characteristic (ROC) curve analysis with corresponding area under the curve (AUC), calibration plots, and decision curve analysis (DCA) to evaluate clinical utility. *P* < 0.05 was considered statistically significant.

## Result

### Definition of prolonged ICU stay

In this cohort study, the length of hospital stay was 3 (1–94) days. Currently, there is no universally accepted standard for defining prolonged intensive care unit (ICU) stay in patients with sepsis-induced coagulopathy (SIC). Based on previous studies [18–20]and relevant statistical methodologies, the third quartile value (5 days) of ICU length of stay for all patients in this cohort was defined as the threshold for prolonged ICU stay.

### Patient characteristics

A total of 3728 patients with SIC were finally included in this study, among which 832 patients were in the prolonged ICU stay group and 2896 patients were in the non-prolonged group. Statistically significant differences (*p* < 0.05) were observed between groups regarding gender, age, weight, BMI, heart rate, DBP, MAP, SpO₂, RBC, WBC, PLT, RDW, INR, neutrophil percentage, lymphocyte percentage, monocyte percentage, SOFA score, and the prevalence of hypertension, T2DM, AKI, HF, use of vasopressors, and use of mechanical ventilation (Table 1).

**Table 1:** Baseline characteristics of ICU LOS for patients with SIC

	Length of stay in the ICU		
Variable	Non-prolonged group	Prolonged group	*P*
	*N* = 2896	*N* = 832	
Female, *n* (%)	1035.0 (35.74%)	336.0 (40.38%)	0.014*
Age	68.0 (58.0–78.0)	67.0 (55.0–77.0)	0.004*
Weight (kg)	80.0 (69.0–94.0)	82.7 (69.0–100.3)	< 0.001*
Height (m)	1.7 (1.6–1.79)	1.7 (1.6–1.8)	0.082
BMI (kg/m^2^)	27.8 (24.3–31.9)	28.7 (24.8–33.9)	< 0.001*
Vital sign			
HR (bpm)	83.0 (75.0–95.0)	89.0 (79.0–105.0)	< 0.001*
SBP (mmHg)	112.0 (99.5–126.0)	114.0 (98.0–133.0)	0.261
DBP (mmHg)	61.0 (53.0–71.0)	62.5 (53.0–74.0)	0.013*
MAP (mmHg)	74.0 (66.0–84.0)	75.0 (65.0–88.0)	0.012*
SPO_2_ (%)	100.0 (97.0–100.0)	99.0(95.0–100.0)	< 0.001*
Temperature (℃)	36.7 (36.4–37.1)	36.8 (36.4–37.2)	0.84
Laboratory parameters			
RBC (× 10^9^/L)	3.2 (2.8–3.7)	3.3 (2.9–3.9)	< 0.001*
WBC (× 10^9^/L)	10.9 (7.5–15.1)	11.4 (7.4–16.8)	0.038*
PLT (× 10^9^/L)	129.0(103.0–154.0)	132.0(100.0–187.0)	0.002*
RDW (%)	14.0(13.2–15.4)	14.7 (13.6–16.1)	< 0.001*
INR	1.5 (1.3–1.6)	1.5 (1.3–1.8)	< 0.001*
Neutrophil percentage (%)	79.1 (72.0–85.2)	81.2 (73.2–87.6)	0.006*
Lymphocyte percentage (%)	12.7 (6.9–19.0)	9.4 (5.2–15.2)	< 0.001*
Monocyte percentage (%)	4.0 (2.4–6.0)	4.4 (2.6–7.0)	< 0.001*
Scores			
SOFA	6(4–8)	8 (5–10)	< 0.001*
GCS	15(14–15)	15 (13–15)	0.094
Underlying disease/comorbidity (n, %)			
Hypertension	1357(46.86%)	313 (37.62%)	< 0.001*
T2DM	839(28.97%)	272 (32.69%)	0.039*
AKI	827 (28.56%)	446 (53.61%)	< 0.001*
HF	668 (23.07%)	290(34.86%)	< 0.001*
Interventions (*n*, %)			
Use of mechanical ventilation	2509 (86.64%)	801 (96.27%)	< 0.001*
Use of vasopressors	1996 (68.92%)	631 (75.84%)	< 0.001*
Clinical outcomes (*n*, %)			
7-day mortality	219 (7.56%)	47 (5.65%)	0.059
14-day mortality	239(8.25%)	122 (14.66%)	< 0.001*
28-day mortality	255 (8.81%)	160 (19.23%)	< 0.001*
90-day mortality	284 (9.81%)	197 (23.68%)	< 0.001*

BMI, body mass index; HR, heart rate; SBP, systolic blood pressure; DBP, diastolic blood pressure; MAP, mean arterial pressure; SpO₂, peripheral capillary oxygen saturation; RBC, red blood cell; WBC, white blood cell; PLT, platelet; RDW, red cell distribution width; INR, international normalized ratio; SOFA, Sequential Organ Failure Assessment; GCS, Glasgow Coma Scale; T2DM, type 2 diabetes mellitus; AKI, acute kidney injury; HF, heart failure

### LASSO regression analysis and predictor screening

**Fig. 3:**
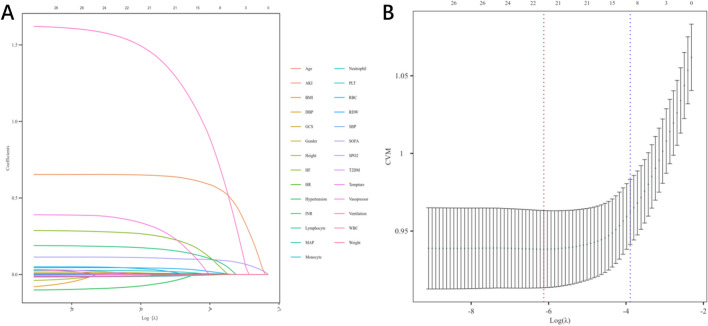
Least absolute shrinkage and selection operator (LASSO) coefficient path plot. **A** Coefficient profiles of variables as a function of log(λ). **B** Cross-validation results of the LASSO logistic regression model. The x-axis represents the log(λ), and the y-axis indicates the mean cross-validation error. The solid line shows the trend of the mean error, and the dashed lines represent the upper and lower limits of the error SHAP interpretable analysis

To mitigate high inter-correlations among variables and prevent model overfitting, LASSO-logistic regression was applied to 23 candidate variables. A tenfold cross-validation was performed using binomial deviance as the evaluation metric, aiming to minimize model deviance. Ultimately, 12 predictors for prolonged intensive care unit (ICU) length of stay were identified: age, white blood cell count, red cell distribution width, international normalized ratio, lymphocyte percentage, monocyte percentage, heart rate, Sequential Organ Failure Assessment (SOFA) score, acute kidney injury, heart failure, use of mechanical ventilation, and use of vasopressors (Fig. 3).

**Fig. 4:**
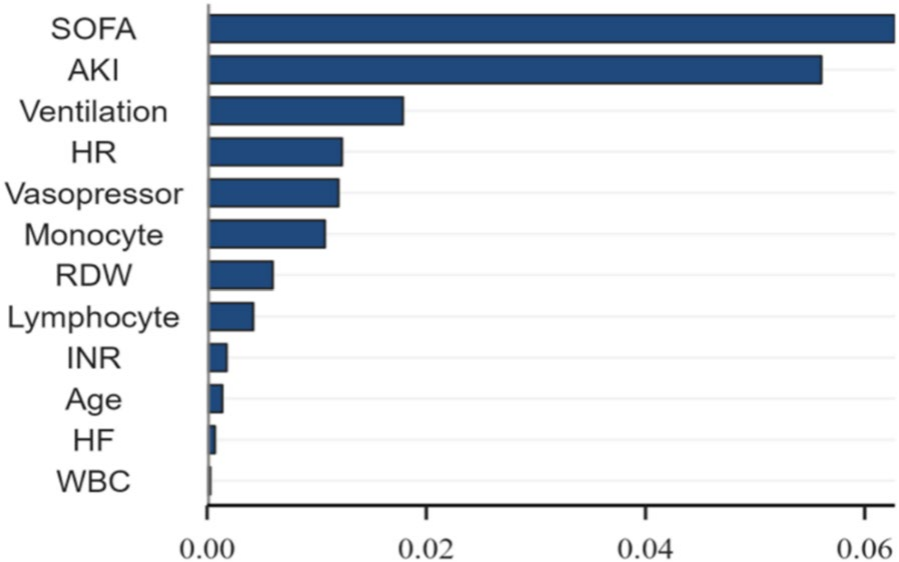
SHAP feature importance plot. SOFA, Sequential Organ Failure Assessment; AKI, acute kidney injury; HR, heart rate; RDW, red cell distribution width; INR, international normalized ratio; HF, heart failure; WBC, white blood cell

SHAP analysis ranked the importance of the 12 predictors as follows: SOFA score, AKI, ventilator used, heart rate, vasopressin used, monocyte percentage, lymphocyte percentage, RDW, INR, age, HF, WBC (Fig. 4).

### Development and evaluation of the nomogram

**Fig. 5:**
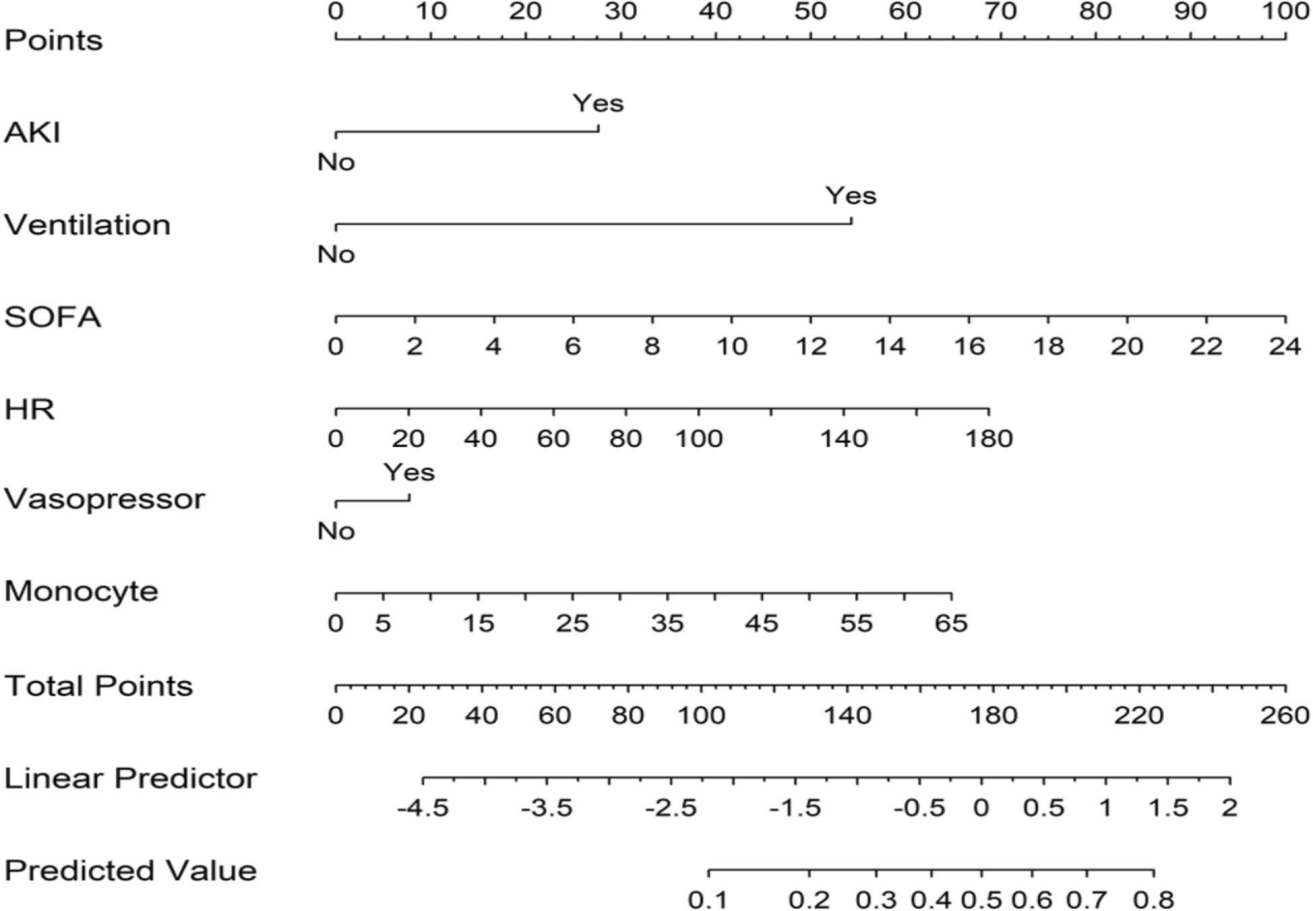
Nomogram model to predict prolonged ICU stay in SIC patients. AKI, acute kidney injury; SOFA, Sequential Organ Failure Assessment; HR, heart rate

To enhance clinical applicability, the nomogram was developed by integrating the top six predictors identified through the SHAP analysis: Sequential Organ Failure Assessment (SOFA) score, heart rate, monocyte percentage, acute kidney injury (AKI), use of vasopressors, and use of mechanical ventilation (Fig. 5), and selecting the variables incorporated into a multivariate logistic regression model to further assess their association with patient outcomes. As shown in Table 2, all included variables were significantly associated with adverse outcomes (*P* < 0.05 for all).

**Table 2:** Multivariable logistic regression of nomogram-included predictors

Variables	*β*	SE	*Z*	*P*	OR (95% CI)
Intercept	−5.19	0.30	−17.05	< 0.01	0.00(0.00–0.00)
AKI	0.81	0.08	9.09	< 0.01	2.25(1.89–2.68)
Ventilation	1.59	0.20	7.96	< 0.01	4.93(3.38–7.44)
SOFA	0.12	0.01	9.15	< 0.01	1.13(1.10–1.16)
HR	0.01	0.00	5.19	< 0.01	1.01(1.00–1.01)
Vasopressor	0.22	0.10	2.26	0.02	1.25(1.03–1.53)
Monocyte	0.03	0.01	2.73	0.01	1.02(1.00–1.05)

AKI, acute kidney injury; SOFA, Sequential Organ Failure Assessment; HR, heart rate

**Fig. 6:**
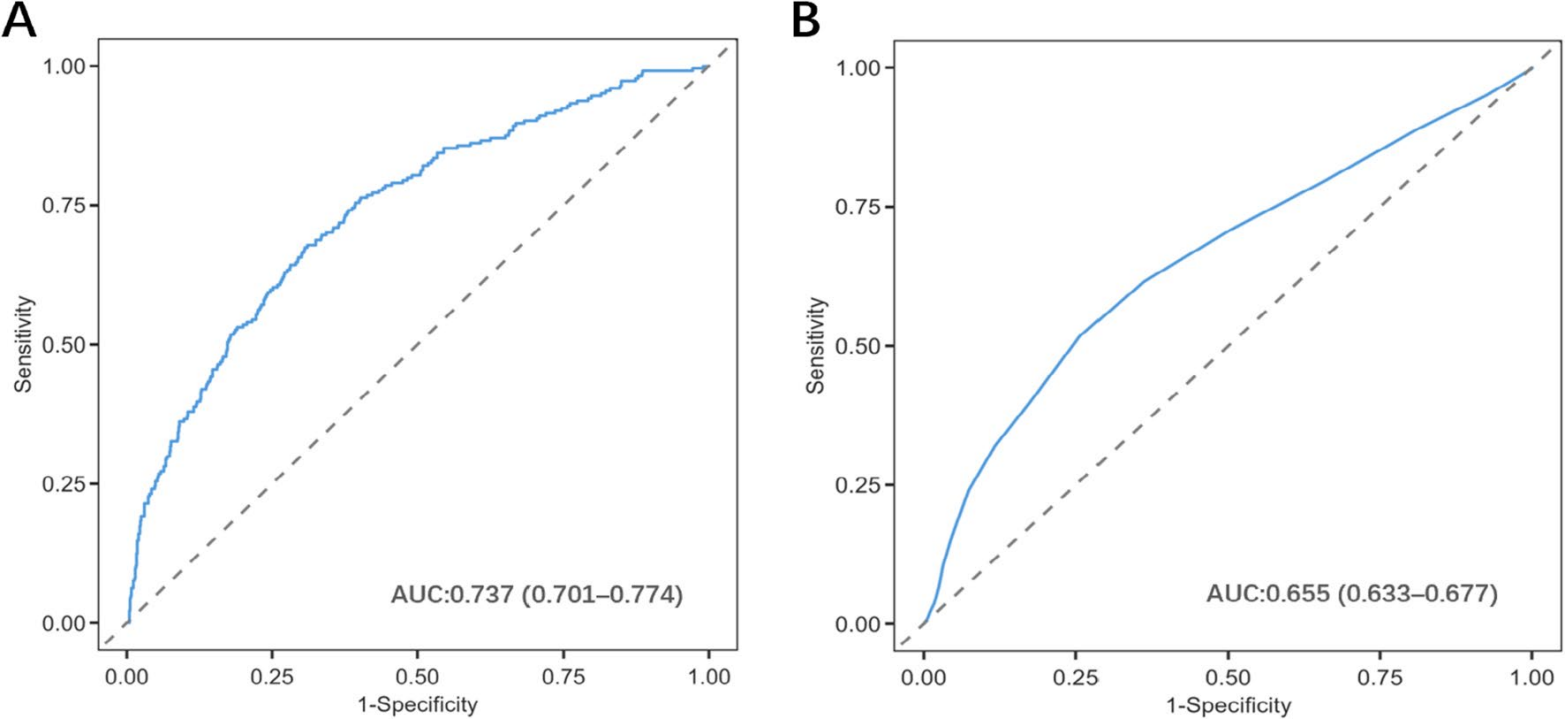
**A** ROC curves of the nomogram to predict prolonged ICU stay in SIC patients. **B** ROC curves of the SOFA to predict prolonged ICU stay in SIC patients

The model demonstrated good discriminatory ability, with an area under the curve (AUC) of 0.737 (95% CI 0.716–0.758) (Fig. 6A). For comparison, a separate predictive model based solely on the SOFA score was also constructed. This model achieved an AUC of 0.655 (95% CI 0.633–0.677) (Fig. 6B). The results indicate that the predictive efficacy of our nomogram model is significantly superior to that of the SOFA score alone.

**Fig. 7:**
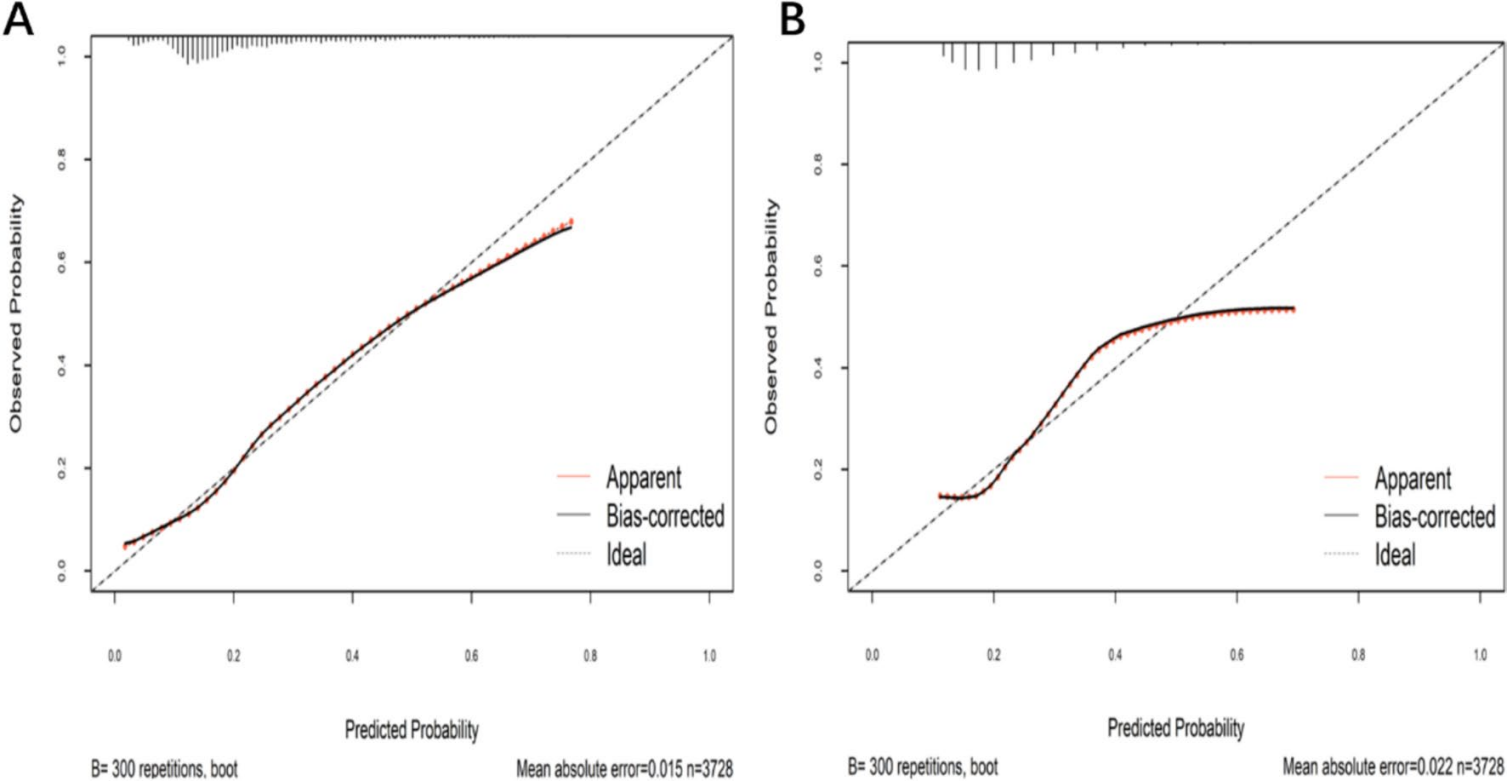
**A** The calibration model of the prolonged ICU stay prediction of the nomogram. **B** The calibration model of the prolonged ICU stay prediction of the SOFA

**Fig. 8:**
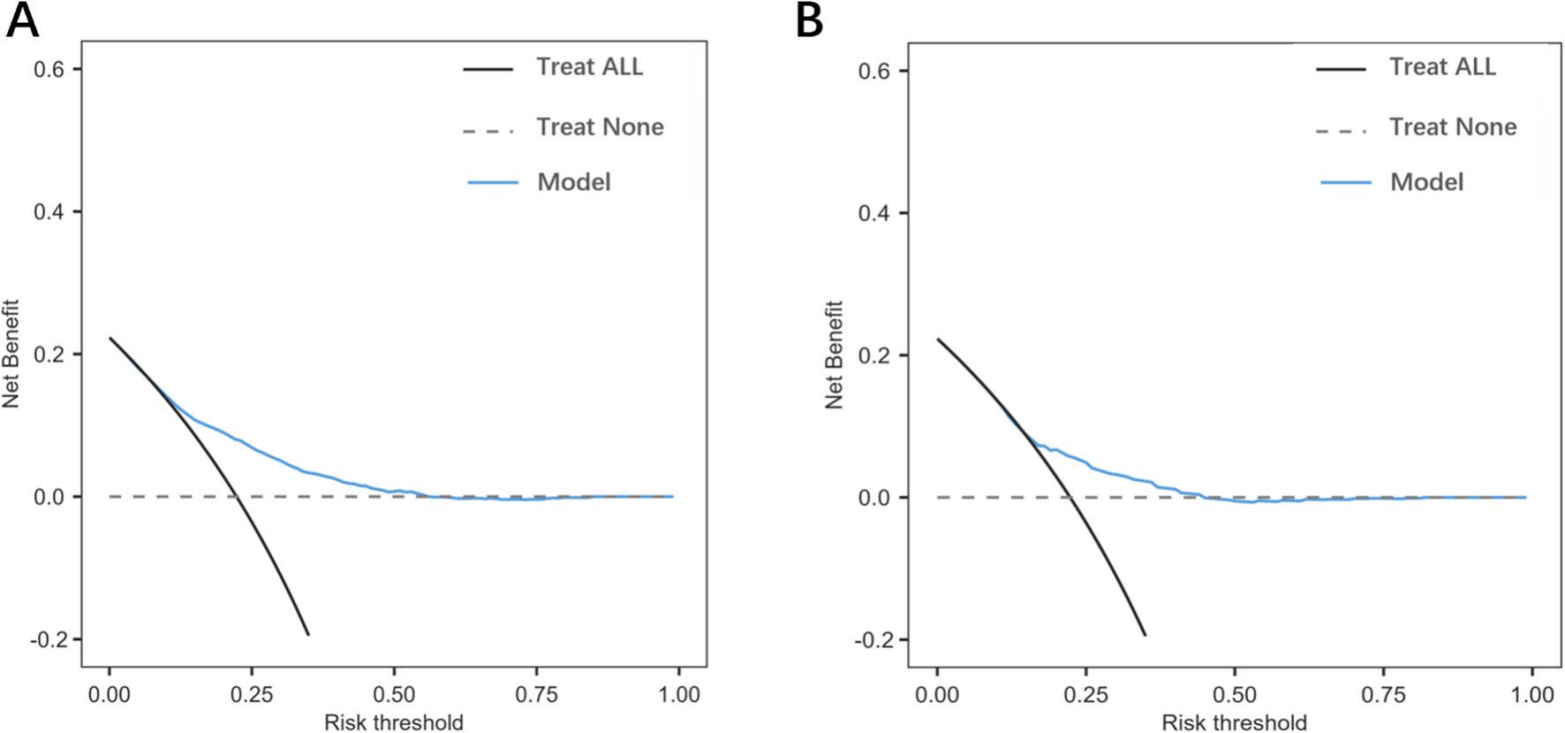
**A** The decision curve analysis of the prolonged ICU stay prediction of the nomogram. **B** The decision curve analysis of the prolonged ICU stay prediction of the SOFA

Furthermore, the calibration curve demonstrated good agreement between predicted probabilities and observed probabilities of prolonged ICU stay (Fig. 7). The bias-corrected calibration curve closely aligned with the ideal reference line, reflecting the model’s good fit to the data. Decision curve analysis (DCA) indicated that the model provides favorable clinical net benefit across a range of threshold probabilities, supporting its clinical utility (Fig. 8).

## Discussion

To our knowledge, this retrospective cohort study is the first to develop a nomogram for predicting prolonged ICU length of stay (LOS) in patients with sepsis-induced coagulopathy (SIC). Leveraging LASSO-logistic regression for feature selection and SHAP (Shapley Additive Explanations) as an interpretable machine learning method, the study provided an explainable analysis by quantifying and ranking feature contributions. Ultimately, six key predictive factors were identified: Sequential Organ Failure Assessment (SOFA) score, heart rate, monocyte percentage, comorbid acute kidney injury (AKI), use of vasopressors, and use of mechanical ventilation. The final model demonstrated favorable discriminative ability (AUC = 0.737) and good calibration, offering a straightforward and clinician-friendly tool for the early assessment of prolonged ICU stay risk in SIC patients.

As the most significant predictor in this study, the Sequential Organ Failure Assessment (SOFA) score serves as a key tool for predicting organ dysfunction [21]. It evaluates parameters across six systems, including hematological function. Reflecting the severity of illness in septic patients [22] and serving as one of the diagnostic criteria for SIC [16], the SOFA score effectively captures the pathophysiological state and clinical presentation of SIC patients. In this cohort study, patients in the prolonged-stay group exhibited significantly higher SOFA scores than those in the non-prolonged group. The research indicates that the SOFA score can predict 28-day mortality in SIC patients [23], with higher scores associated with worse prognosis. Furthermore, an elevated SOFA score signifies greater disease severity and a higher degree of dysregulation in both the coagulation and inflammatory systems [24]. The mechanisms underlying coagulation dysfunction in SIC patients are highly complex, and the current evidence regarding anticoagulation therapy for septic patients with varying degrees of coagulation disorder remains inconclusive [25]. This complexity adds to the therapeutic challenge, necessitating a longer period for treatment, recovery, and clinical observation.

Simultaneously, as one of the most fundamental treatment strategies in the ICU, mechanical ventilation serves as the primary method for respiratory management in intensive care units, playing an irreplaceable role in maintaining organ function in sepsis. Sepsis is characterized by a dysregulated host immune response to infection, leading to coagulopathy. This resulting coagulopathy further enhances inflammatory activity, potentially causing organ dysfunction and exacerbating lung injury [26], ultimately leading to respiratory dysfunction. This study observed a significant positive correlation between the duration of respiratory support and ICU length of stay in patients with sepsis-induced coagulopathy (SIC) who received mechanical ventilation. Research indicates that early mechanical ventilation is associated with worse patient prognosis [27]. Therefore, as a treatment modality, its duration or the difficulty of weaning from ventilation can reflect the complexity of the patient's condition. However, it must also be noted that mechanical ventilation itself is an invasive therapy, and its management strategies (such as tidal volume and positive end-expiratory pressure settings) may also influence patient outcomes [28]. Future studies need to further distinguish the impact of SIC severity itself from that of mechanical ventilation on clinical outcomes.

In this study, the incidence of acute kidney injury (AKI) was significantly higher in the prolonged-stay group, nearly doubling that of the non-prolonged group. Sepsis, as a critical condition involving immune and inflammatory responses, is a leading cause of AKI. Due to excessive activation of coagulation and deficits in anticoagulant and fibrinolytic regulatory systems [29], this can increase the severity of patient prognosis and mortality, which may represent a significant contributing factor in the development of AKI. Research indicates that DIC is closely associated with the risk of AKI, even after adjusting for severity and other related factors [30]. Although there is currently no definitive study on the relationship between SIC and AKI, both sepsis and DIC have been confirmed to be associated with AKI, and most patients with these conditions have bad prognoses. These findings collectively suggest that SIC, as a critical intermediate stage in the progression of sepsis and DIC, likely plays a role in driving the pathogenesis of AKI. One study indicates that platelets, a diagnostic criterion for SIC, can mediate acute renal failure [31]. Therefore, the occurrence of AKI during the ICU stay of SIC patients may serve as a risk factor for bad prognosis, significantly extending their ICU length of stay. However, further direct research evidence is still needed to substantiate the direct relationship between SIC and AKI.

During the progression of sepsis and sepsis-induced coagulopathy (SIC), an elevated heart rate is one of the core manifestations of the body’s compensatory response. When a patient develops sepsis, the inflammatory reaction leads to vasodilation and a reduction in systemic vascular resistance, resulting in hypotension and decreased cardiac output [32]. To compensate, the body increases the heart rate to enhance cardiac output and ensure adequate tissue perfusion. As one of the most easily measurable clinical parameters in the hospital, heart rate (HR) also serves as a crucial indicator of shock and sensitively reflects the functional state of blood circulation [33].

More and more evidence suggests that systemic inflammatory responses play a significant role in heart rate variations in conditions such as sepsis [34]. Particularly in patients with sepsis and SIC, the vicious cycle between the coagulation and inflammatory systems may further exacerbate microcirculatory dysfunction, making compensatory tachycardia more pronounced and difficult to correct [35]. This phenomenon can be utilized for the early assessment of disease severity in SIC patients. In this cohort study, the heart rate was higher in the prolonged-stay group than in the non-prolonged group, and the use of vasoactive drugs was also more frequent in the prolonged-stay group. Vasoactive drugs are commonly employed in patients with hypotensive shock. If septic patients, including those with SIC, develop shock, they face a high mortality risk [26], and their condition is often difficult to correct through fluid resuscitation alone. In such cases, vasoactive drug intervention is necessary to improve tissue and organ perfusion, reverse tissue ischemia, and hypoxia. The Surviving Sepsis Campaign 2021 guidelines also recommend the use of vasopressors for septic patients in a state of hypotension [26]. In summary, the early use of vasoactive drugs in SIC patients may be associated with the severity of coagulation dysfunction. Early administration of vasoactive drugs in SIC patients often indicates a worse prognosis, providing a potential integrative assessment approach for the early identification of high-risk patients.

Recent studies suggest that monocytes, as key cellular components linking innate immunity and the coagulation system, may play a significant role in the inflammatory–coagulation response and can modulate coagulation function [36]. In sepsis, depletion of monocytes shortens thrombin peak formation time and elevates the levels of certain coagulation factors [37], indicating that an increase in monocytes is associated with a higher likelihood of progressing to sepsis. Another possible explanation is that patients with sepsis-induced coagulopathy (SIC) exhibit more pronounced immune dysfunction and an overactivated inflammatory response [38], which is closely linked to worse prognosis in SIC patients. The elevated monocyte levels observed in this study were associated with the prolonged-stay group. In clinical practice, monocyte counts offer the advantage of being simple and readily obtainable, facilitating the provision of early information on disease progression in SIC patients, thereby holding considerable clinical utility. In the future, more comprehensive and multidimensional models could be developed by incorporating different monocyte phenotypes or specific inflammatory mediators.

Although the nomogram achieved an AUC of 0.737, the strength of this study’s model lies in its concise set of predictors, which includes variables such as SOFA score, heart rate, monocyte percentage, comorbid acute kidney injury (AKI), use of vasopressors and use of mechanical ventilation—all readily obtainable during routine ICU care. The findings of this study preliminarily elucidate the association between prolonged hospital stay in sepsis-induced coagulopathy (SIC) patients and these commonly measured clinical variables. From a research perspective, a model constructed using routine clinical variables offers greater potential for clinical applicability. Compared to traditional disease severity scores like APACHE II or SAPS II, our model is more targeted, enabling early identification of this high-risk SIC subgroup. Furthermore, while other sepsis models primarily focus on mortality as the clinical endpoint, this study investigates prolonged ICU length of stay. This outcome not only facilitates the early identification and intervention for high-risk patients, but also reveals that long-term mortality in the prolonged-stay group is significantly higher than in the non-prolonged group. This association provides an early warning signal for patient survival prognosis to some extent, while also promoting healthcare resource efficiency.

Finally, we acknowledge several limitations in this study. Firstly, utilizing the MIMIC database, the retrospective nature of the research inherently precludes control over or precise assessment of certain critical treatment details and clinical decision-making processes. Potential confounding factors that could not be specifically measured include, for example, the specific strategies for vasopressors administration and ventilation management. Secondly, there is currently no authoritative definition for prolonged length of stay specifically in SIC patients. The definition adopted in this study was based on the distribution within the present cohort, which carries a risk of bias. Thirdly, as a single-center retrospective study, the generalizability of the findings may be limited. The results could be influenced by various factors such as individual racial differences, types of infection, and treatment protocols, potentially affecting the robustness of the conclusions. Lastly, certain factors not included in the model, such as the site of infection and the patient's pre-existing physical condition, might influence the accuracy of predicting prolonged ICU stay. Future research should focus on validating and refining the model through multi-center, prospective studies. This approach would facilitate further variable screening and model optimization, thereby enhancing its robustness and clinical utility.

## Conclusion

Risk factors for prolonged LOS in patients with SIC included: increased SOFA score, elevated heart rate, higher monocyte percentage, occurrence of AKI, use of vasopressors, and use of mechanical ventilation. By integrating these readily available clinical indicators into an intuitive nomogram, we have developed a practical risk assessment tool for clinicians. This tool aids in the identification of SIC patients at risk for prolonged hospitalization. Furthermore, our analysis revealed that prolonged LOS was significantly associated with increased long-term mortality. The application of this predictive model may ultimately contribute to reducing ICU length of stay and improving patient prognosis.

## Data Availability

The data supporting this study are available from the MIMIC-IV database (https://mimic-iv.mit.edu/). Access requires completion of the required training and compliance with the data use agreement.
